# Brain Injury and Neurodevelopmental Outcome in Survivors After Spontaneous Single Fetal Demise in Monochorionic Twins: A Systematic Review and Meta‐Analysis

**DOI:** 10.1111/1471-0528.70084

**Published:** 2025-11-12

**Authors:** Mathies Rondagh, Lotte C. M. Zwinkels, Jeanine M. M. van Klink, Linda S. de Vries, Sylke J. Steggerda, Femke Slaghekke, E. J. T. (Joanne) Verweij, Monique C. Haak, Sophie G. Groene, Enrico Lopriore

**Affiliations:** ^1^ Division of Neonatology, Department of Pediatrics, Willem‐Alexander Children's Hospital Leiden University Medical Center Leiden the Netherlands; ^2^ Division of Fetal Medicine, Department of Obstetrics Leiden University Medical Center Leiden the Netherlands

**Keywords:** acute exsanguination, brain injury, monochorionic twins, neurodevelopmental outcome, single fetal demise

## Abstract

**Background:**

Monochorionic (MC) twins are at risk of acute exsanguination after single fetal demise (sFD) due to their shared placental circulation, which may result in sequelae for survivors.

**Objectives:**

To evaluate the prevalence of ante‐ and postnatal brain injury and long‐term neurodevelopmental impairment (NDI) in co‐twins after sFD. Secondary outcomes were the prevalence of termination of pregnancy (TOP), neonatal death (NND) and potential risk factors for brain injury.

**Search Strategy:**

PubMed, Embase, Scopus and Web of Science were searched to identify relevant studies in October 2024.

**Selection Criteria:**

Studies reporting MC twin pregnancies with spontaneous sFD. Studies with selective feticide, twin reversed arterial perfusion sequence, twin anaemia–polycythaemia sequence, congenital anomalies, higher‐order multiple pregnancies, fetoscopic laser surgery and double fetal demise were excluded.

**Data Collection and Analysis:**

Systematic review and meta‐analysis were performed following the PRISMA and MOOSE guidelines.

**Main Results:**

Thirteen studies involving 311 survivors after sFD were included. The prevalence of TOP, NND, brain injury and NDI was 3% (95% CI: 0%–7%), 6% (95% CI: 0%–16%), 27% (95% CI: 18%–37%), 6% (95% CI: 3%–11%), respectively. The median GA at birth in survivors with brain injury was 29 weeks (IQR 27.7–34.1) compared to 36 weeks (IQR: 32.3–37.0) in the overall group of survivors.

**Conclusions:**

Brain injury occurs in one in four survivors and is associated with lower GA at birth, suggesting a double‐hit injury due to a combination of exsanguination and (severe) prematurity. NDI occurs in one in 20 survivors, compared to two‐thirds of those with brain injury.

**Trial Registration:**

PROSPERO number: CRD42024608912

## 1 Background

Monochorionic (MC) twin pregnancies are associated with an increased risk of perinatal morbidity and mortality due to a shared placental circulation, predisposing them to complications including twin‐twin transfusion syndrome (TTTS) and selective fetal growth restriction (sFGR) [1, 2, 3, 4]. TTTS is caused by an imbalanced blood flow, resulting in polyhydramnios and polyuria in the recipient twin and oligohydramnios and oliguria in the donor twin. sFGR arises from unequal placental sharing, leading to significant intertwin growth discordance [2]. These complications can lead to single fetal demise (sFD) or double fetal demise (dFD) [2, 3, 4]. sFD occurs in 15% of MC pregnancies compared to 3% in dichorionic pregnancies [5].

Following sFD, survivors are at risk of fetal demise or neonatal death (NND), brain injury and neurodevelopmental impairment (NDI) [6, 7, 8]. In the past, brain injury after sFD was thought to result from thrombo‐embolic processes due to passage of thrombotic material through the vascular anastomoses. However nowadays the widely accepted hypothesis is that this results from sudden exsanguination, leading to hypovolemic shock, severe fetal anaemia, hypoxic–ischemic brain injury in the surviving co‐twin [3, 9].

The most recent systematic review and meta‐analysis (published in 2018) in MC twins with sFD reported abnormal antenatal and postnatal brain imaging findings in 20% (95% CI: 13%–31%) and 43% (95% CI: 33%–56%) of the cases, respectively, and NDI in 29% (95% CI: 19%–43%) of the survivors [8]. However, this systematic review, was based on a limited number of small retrospective studies and case series, with considerable heterogeneity. Comprehensive data on the aetiology of spontaneous sFD, incidence and type of brain injury, and long‐term neurodevelopmental outcome of MC twin pregnancies, therefore, remain limited and are currently outdated [7, 8]. In the past few years, several new and larger cohort studies in MC twins with sFD were published, sharing additional data and shedding new light on this important topic. We therefore performed an up‐to‐date systematic review to guide clinical decision making, support parental counselling and identify gaps in the literature.

This systematic review aimed to evaluate the prevalence of brain injury both antenatal and postnatal, and long‐term NDI in survivors after sFD in MC twin pregnancies. In addition, we studied the prevalence of termination of pregnancy (TOP), NND, and potential risk factors for brain injury including GA at sFD, GA at birth, and concomitant presence of MC pregnancy complications including TTTS and sFGR.

## 2 Methods

### 2.1 Study Design

This systematic review was conducted in accordance with the Preferred Reporting Items for Systematic Reviews and Meta‐Analyses (PRISMA) and Meta‐analyses and systematic reviews of Observational Studies' (MOOSE) guidelines [10, 11]. The protocol was registered in PROSPERO (CRD42024608912, https://www.crd.york.ac.uk/PROSPERO) [12].

### 2.2 Data Sources and Search Strategy

A systematic literature search was conducted on October 31, 2024, in the Embase, PubMed, Scopus and Web of Science database to identify relevant studies. The search strategy included the terms ‘monochorionic twins’ and ‘fetal demise’, along with various synonyms as both free‐text keywords and MeSH terms (Appendix S1). To ensure a comprehensive search scope, outcome‐related terms were not included in the search strategy. The search strategy was developed in consultation with an information specialist from the Leiden University Medical Center Walaeus library. The reference lists of the selected studies were manually reviewed to identify potentially relevant articles.

### 2.3 Study Selection and Data Extraction

All relevant articles were evaluated for eligibility by screening their titles and abstracts. Articles that met the inclusion criteria were subjected to full‐text screening. Inclusion criteria were studies reporting at least three cases of MC twin pregnancies complicated by sFD occurring at ≥ 12 weeks of gestation with publication from 1999 onwards. We excluded studies that did not distinguish outcomes of MC twins with sFD, studies involving sFD after fetoscopic laser surgery, selective feticide, twin reversed arterial perfusion (TRAP) sequence, congenital or chromosomal anomalies, higher‐order multiples, twin anaemia‐polycythemia sequence (TAPS), or dFD. We excluded TAPS cases since a recent multicentre study showed that sFD in TAPS does not lead to brain injury or death in the survivors as the minuscule anastomoses preclude acute exsanguination [13] Congenital anomalies were excluded because outcome is primarily attributable to the anomaly rather than to MC‐related complications and/or acute exsanguination after sFD. Editorials, letters, reviews, case reports, case series (*n* ≤ 3), studies not written in English, or when the full text was unavailable, were excluded from this systematic review. Two reviewers (M.R. and L.C.M.Z.) assessed the search results. In case of disagreement, a third author (E.L.) was introduced to reach a consensus.

### 2.4 Outcome Measures and Definitions

We recorded fetal demise of the co‐twin due to TOP. NND was defined as death of a live‐born infant within the first 28 days after birth. Preterm birth was registered and classified into three categories: moderate (32–36 weeks), very preterm (28 to < 32 weeks) and extremely preterm (< 28 weeks). Brain injury was classified as antenatal or postnatal injury, and defined as cystic periventricular leukomalacia (cPVL), multicystic encephalomalacia, white matter (WM) injury (unspecified), cortical malformations, intraventricular haemorrhage (IVH), infarction, volume loss and/or severe ventriculomegaly (VM), or remained unknown on fetal and/or postnatal ultrasound or MRI [14, 15, 16]. Lastly, we recorded the presence of any form of NDI, including all long‐term sequelae, cognitive and/or motor developmental delay and cerebral palsy (CP). The primary outcome was the prevalence of brain injury (either antenatal or postnatal) and the prevalence of NDI at follow‐up. Secondary outcomes included the type of brain injury, the type of NDI, the prevalence of sFD, TOP and NND. We evaluated the association between sFD and brain injury including potential risk factors such as GA at sFD, GA at birth and concomitant presence of MC pregnancy related complications.

### 2.5 Assessment of Risk of Bias

The Newcastle–Ottawa Scale was used to assess the quality of non‐randomised studies, focusing on selection, comparability and outcome [17]. Articles were classified as good, fair, or poor quality (Appendix S2). Two authors (M.R. and L.C.M.Z.) independently evaluated methodological quality, resolving discrepancies through discussion until consensus was reached.

### 2.6 Statistical Analysis

Descriptive statistics are presented as mean ± standard deviation (SD), median (interquartile range [IQR]) or 95% confidence interval (CI), for continuous variables and as percentages for categorical variables. For missing statistical data, means and IQR were computed based on the available relevant cases. When data were unavailable in the included studies, the primary authors of the included studies were contacted to provide additional information. The results of the mixed effects logistic regression models were expressed as odds ratios (ORs) and 95% confidence intervals (CIs). Pooled proportions were estimated using fixed‐effect and random‐effects models. Wilson score confidence intervals were used for individual study estimates. Heterogeneity was assessed using Cochran's *Q*‐test (*p* < 0.05 indicating significance), *I* ^2^ statistic (≥ 50% considered substantial heterogeneity) and tau‐squared (*τ* ^2^) to estimate between‐study variance [18]. When heterogeneity was high, a random‐effects model was used. All analyses were conducted using RStudio software (version R 4.2.2, Boston, Massachusetts, USA) with statistical significance set at *p* < 0.05. Egger's test for funnel plot asymmetry was performed to assess publication bias, using a linear regression model of effect sizes against their standard errors (Figure S1).

## 3 Results

### 3.1 Study Selection

**FIGURE 1:**
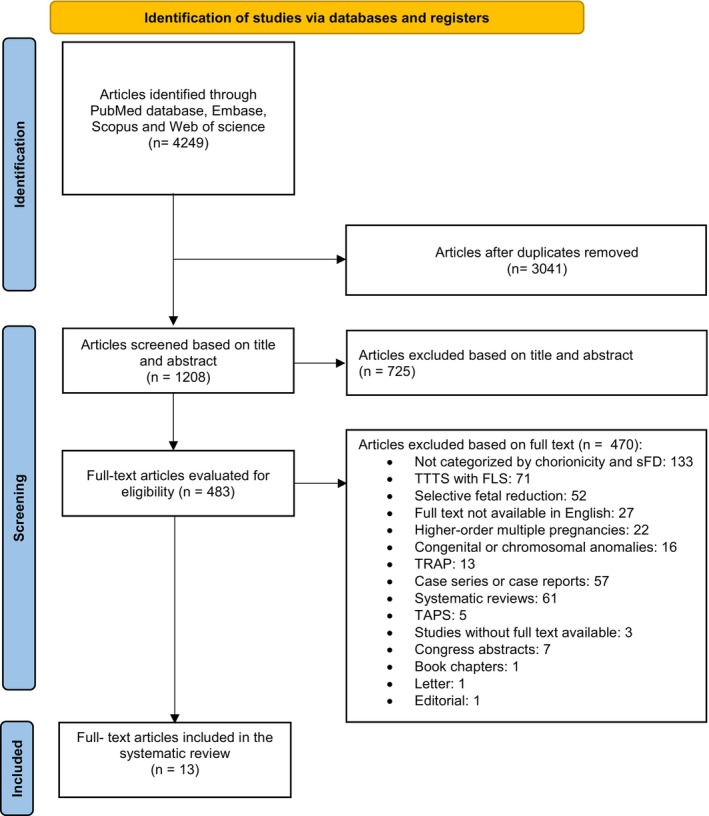
Flow diagram. FLS, fetal laser surgery; sFD, single fetal demise; TAPS, twin anaemia‐polycythaemia sequence; TRAP, twin reversed arterial perfusion sequence; TTTS, twin‐to‐twin transfusion syndrome.

The systematic literature search identified 4249 articles in PubMed, Embase, Scopus and Web of Science. After removal of 3041 duplicates, we screened 1208 article based on title and abstract. Of these, 725 were excluded during the screening based on the title and abstract as they did not meet the inclusion criteria. The remaining 483 full‐text articles were evaluated and 470 articles were excluded for various reasons, as outlined in the flowchart (Figure 1). Ultimately, 13 studies met the eligibility criteria and were included in this systematic review.

### 3.2 Quality Assessment

The quality of the included studies was evaluated and categorised using the Newcastle–Ottawa scale. Out of the 13 studies, 12 (93%) were rated as ‘good quality’, one (7%) as ‘fair quality’ and none as ‘poor quality’. The minimum and maximum scores attained by the studies were 6 and 9 points, respectively (Table S1). The lowest score was attributed to the absence of clearly defined selection criteria, inadequate comparability and a lack of detailed follow‐up criteria. None of the studies were excluded based on the quality assessment.

### 3.3 Study Characteristics

Included studies were published between 1999 and 2023, with study periods extending from 1980 to 2020 (Table S2). The majority of these studies (*n* = 11) were retrospective observational cohort studies, while two were prospective observational cohort studies [19, 20]. In total, 311 survivors from MC twin pregnancies complicated by sFD were evaluated. Individual study sample sizes ranged from 5 to 77 infants. The largest cohort was a recent study published by Lanna et al. [4, 21]. While most studies included general populations of MC twins with sFD, Conte et al. exclusively investigated cases involving brain injury [22].

### 3.4 Clinical Characteristics

**FIGURE 2:**
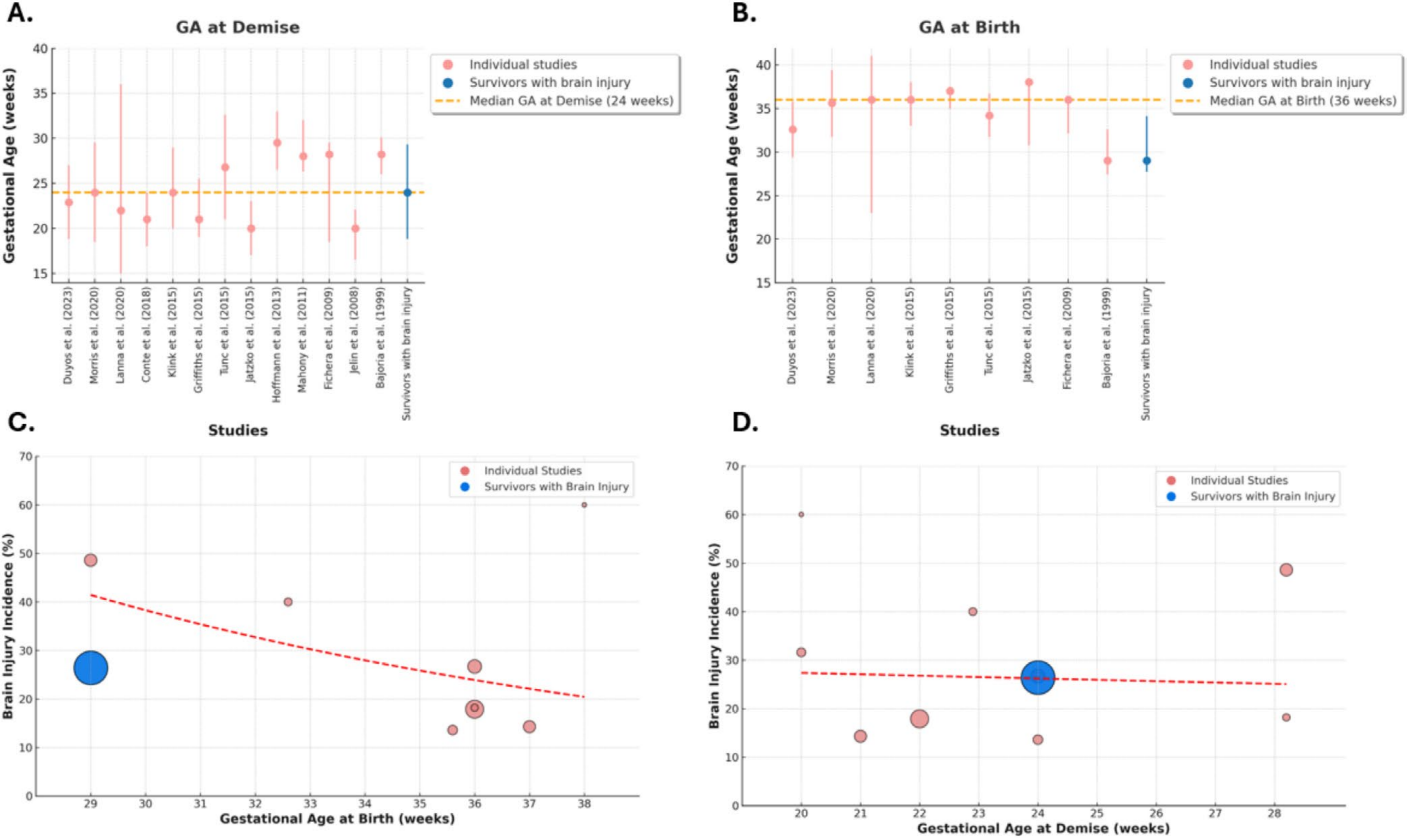
GA at demise, birth and brain injury across studies. (A) Shows the gestational age (GA) at demise, and (B) shows the GA at birth across the included studies. The circles represent median or mean GA, with error bars indicating the interquartile range (IQR), standard deviation (SD) or range. The overall median GA for survivors with brain injury are represented in dark red. (C) Illustrates the prevalance of brain injury in relation to gestational age at birth. Each bubble represents an individual study, with the bubble size corresponding to the study population. (D) Illustrates the prevalance of brain injury in relation to study population. The red dashed line represents the trendline.

The mean or median GA at sFD ranged from 20 to 30 weeks among the included studies (Figure 2) [3, 4, 19, 20, 21, 22, 23, 24, 25, 26, 27, 28, 29]. Preterm delivery was described in 10 studies, with mean or median GA at birth ranging from 29 to 38 weeks [3, 4, 19, 20, 21, 23, 24, 25, 27, 29]. Six studies classified preterm deliveries as extreme preterm, with proportions ranging from 0% to 27%. However, most cases were categorized as either very preterm (0%–43%) or moderate preterm (20%–73%, Table S3) [19, 21, 23, 24, 27, 29]. The aetiology of sFD was described in 10 studies, although it remained unknown in 41% (103/250) of cases. TTTS was presumed the most frequent cause, in 38% (96/250, range 15%–67%) while sFGR was presumed the second most frequent cause in 15% (38/250, range 3%–27%) of cases [3, 4, 20, 22, 23, 25, 26, 27, 28, 29]. Other less frequent causes included placental complications and cord entanglement in monoamniotic twins. Treatment with IUTs to treat fetal anaemia after sFD was reported in 17% (33/191, range 0%–54%, *n* = 6 studies) of cases [3, 4, 20, 23, 27, 29].

### 3.5 Termination of Pregnancy (TOP)

**FIGURE 3:**
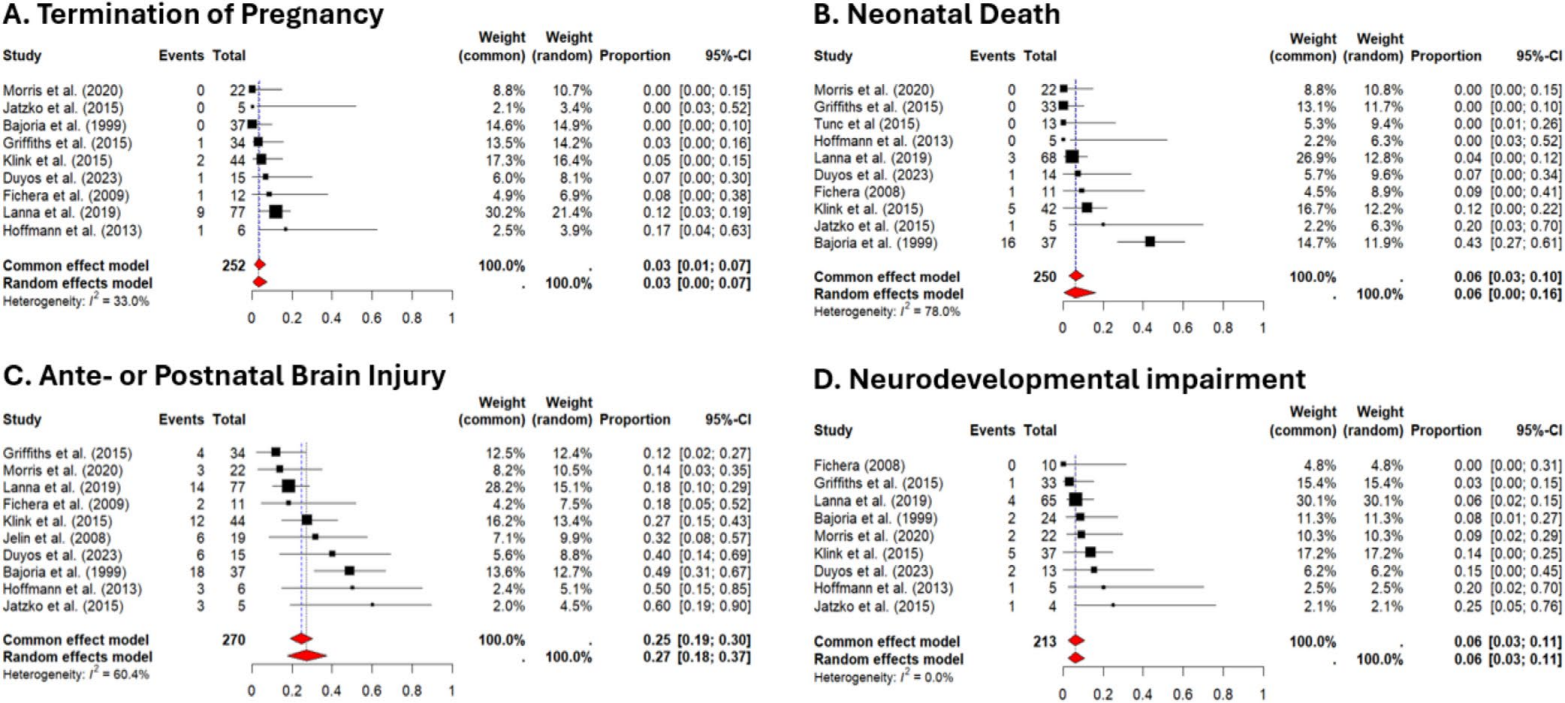
Forest plots of pooled proportions of TOP, NND, brain injury and NDI. Forest plots displaying the pooled proportions of termination of pregnancy (A), neonatal death (B), brain injury (C) and NDI (D) across included studies. Each study is represented by a grey square, with the size of the square proportional to the study weight. The horizontal lines indicate 95% confidence intervals (CIs) for each study estimate. The vertical dashed line represents the pooled estimate. The diamond at the bottom of each plot represents the overall effect size with its corresponding 95% CI, calculated using both the fixed‐effect model and random‐effects model. Heterogeneity statistics includes *I* ^2^.

Nine studies reported the number of TOP following sFD of the co‐twin [3, 4, 19, 20, 21, 23, 24, 27, 29]. The pooled proportion of TOP was 3% (95% CI: 1%–7%), with moderate heterogeneity (*I* ^2^ = 33%, *p* < 0.001, Figure 3A). The highest proportion was 17% (95% CI: 4%–63%), while four studies reported no cases of TOP [19, 20, 21, 29].

### 3.6 Neonatal Death (NND)

Ten studies reported the number of NND following sFD of the co‐twin [4, 19, 20, 21, 23, 24, 25, 27, 29]. The pooled proportion of NND, was 6% (95% CI: 0%–16%, Figure 3B), with substantial heterogeneity (*I* ^2^ = 78%, *p* < 0.001). The highest reported proportion was 43% (95% CI: 27%–61%), while three studies observed no cases of NND [19, 20, 24, 25].

### 3.7 Antenatal or Postnatal Brain Injury

Eleven studies investigated the prevalence and/or type of brain injury in survivors after sFD.

However, the study by Conte et al. was excluded from the evaluation of brain injury prevalence, as it included only co‐twin survivors with brain injury. Fetal ultrasound and/or MRI (2–4 weeks post‐sFD or based on fetal ultrasound abnormalities) were performed in 92% (12/13) of studies. Neonatal ultrasound and/or postnatal MRI were conducted in 62% (8/13). In total, 26% (71/270) of cases (*n* = 10 studies) showed antenatal (13%, 34/270) or postnatal brain injury (6%, 16/270, Table S4) [3, 4, 19, 20, 21, 23, 24, 27, 28, 29]. The reported prevalence of brain injury varied significantly across studies, ranging from 12% to 60%, with the highest number of cases observed in the study by Bajoria et al. [29] The pooled proportion of antenatal or postnatal brain injury, was 27% (95% CI: 18%–37%, *n* = 10 studies, Figure 3C), with significant heterogeneity (*I* ^2^ = 60%, *p* = 0.01).

### 3.8 Neurodevelopmental Impairment

NDI was prevalent in 8% (18/213) of the survivors after sFD. Although follow‐up duration was not consistently reported, when specified, it ranged from 12 months to 5 years [3, 4, 19, 20, 21, 23, 24, 27, 29]. The pooled proportion of NDI was 6% (95% CI: 3%–11%, *n* = 9 studies, Figure 3D), with no observed heterogeneity (*I* ^2^ = 0%, *p* = 0.53). The highest proportion of NDI was observed in a small retrospective study (*n* = 5) by Jatzko et al., with 25% of survivors affected (95% CI: 5%–76%) [21]. Fichera et al. was the only study that reported no survivors of NDI [27].

### 3.9 Risk Factors for Survivors With Brain Injury

**FIGURE 4:**
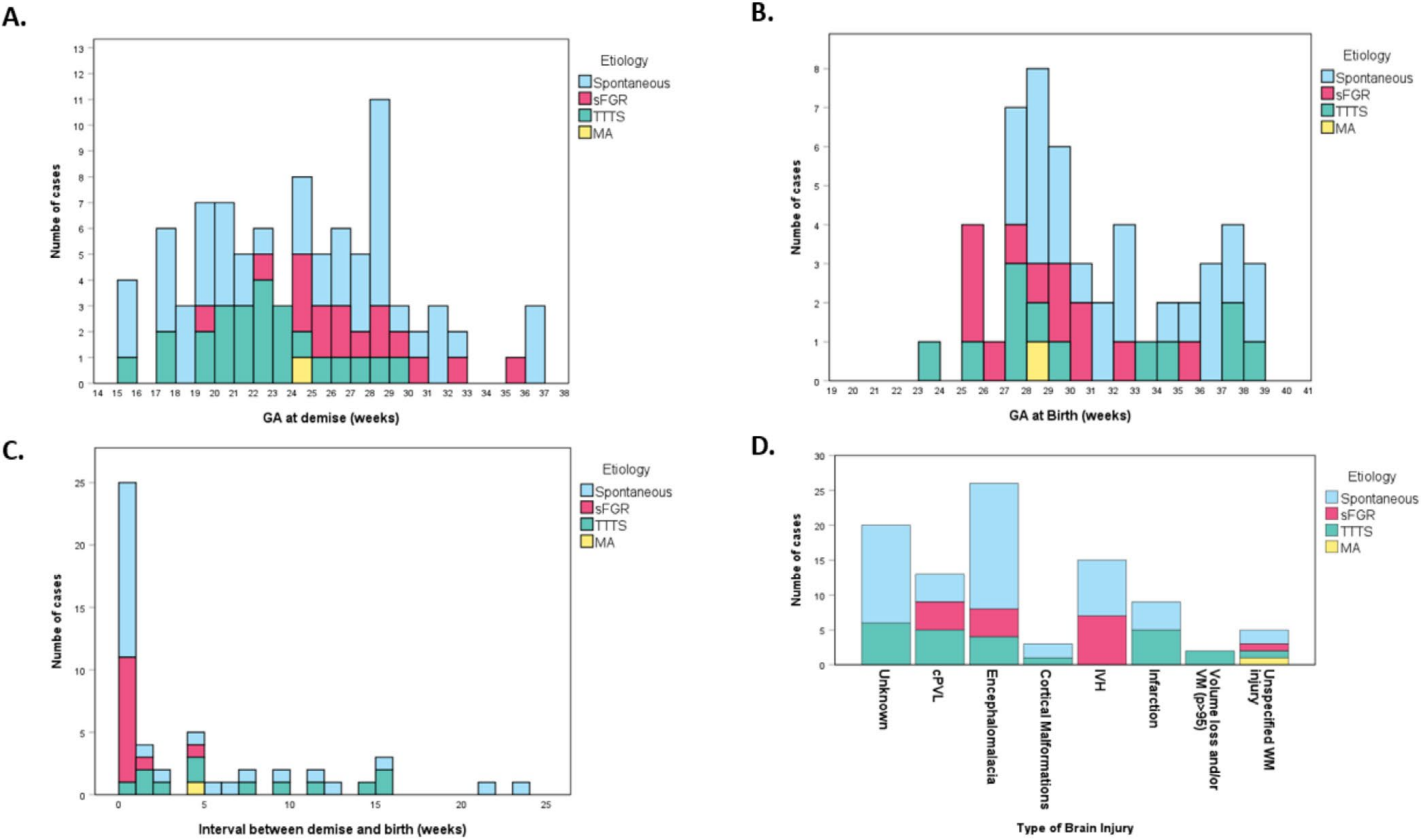
Overview of survivors of MC twin pregnancies after sFD with brain injury. Histograms of all survivors of MC twin pregnancies after sFD with brain injury, categorised by aetiology. (A) Gestational age at demise. (B) Interval between fetal demise and birth. (C) Gestational age at birth of the surviving co‐twin. (D) Types of brain injury in survivors. Colours represent different aetiologies: Spontaneous (blue), sFGR (pink), TTTS (green) and MCMA (yellow).

This systematic review extracted specific data on antenatal and postnatal characteristics of all 92 survivors after sFD with brain injury from 11 studies, including Conte et al. (*n* = 21, Table S5) [3, 4, 20, 21, 22, 23, 24, 27, 28, 29]. Median GA at sFD was 24 weeks (IQR 20.2–28.2, *n* = 89) of survivors with brain injury. In three infants the GA at sFD was not reported. The overall median GA at birth was 29 weeks (IQR 27.7–34.1, *n* = 51, Figure 4), which was lower than the median GA in the overall population of MC pregnancies with sFD (36 weeks, IQR: 32.3–37.0). Of the 50 cases with a reported GA at birth, 14% (7/50) were born term, 24% (12/50) were moderate preterm, 36% (18/50) were very preterm and 26% (13/50) were extremely preterm. In cases where the time between sFD and birth was reported, 56% (28/50) of survivors were delivered within less than 1 week, while 34% (17/50) were delivered on the same day. In 57% (52/92) cases with brain injury no cause of the sFD was identified. TTTS was the presumed cause in 26% (24/92), followed by sFGR in 17% (16/92). A total of 71 cases were initially considered in which the timing of brain injury was reported. However, to avoid selection bias, the study by Conte et al. was excluded, as it only included cases with antenatal brain injury [22]. Following this exclusion, 50 cases remained. Of these, 34 (68%) exhibited antenatal brain injury, while 16 (32%) had postnatal brain injury. The following injuries were related to prematurity postnatally: PVL (*n* = 10), unspecified white matter injury (*n* = 3), infarction (*n* = 1) and IVH (*n* = 2). However, most studies did not report the longitudinal progression or the occurrence of multiple types of brain injury in cases with combined ante‐ and postnatal brain injury. Brain injury was classified from most frequent to less frequent based as follows: cystic encephalomalacia (27%; 25/92) [3, 4, 21, 22, 23, 24, 28], IVH (16%; 15/92) [3, 21, 23, 29], cPVL (14%; 13/92) [3, 4, 23, 29], infarction (10%; 9/92), unspecified white matter injury (5%; 5/92) [4, 22, 23, 27]; cortical malformations (3%; 3/92) [4, 28] and cases with volume loss and/or severe ventriculomegaly (2%; 2/92) [4]. In 22% (20/92) there were insufficient details of brain injury for classification [20, 22, 24, 28, 29]. Neurodevelopmental outcome was reported in 51% (29/57) survivors with brain injury. Among these, 31% (9/29) developed CP, 31% (9/29) NDI that was not further specified and 38% (11/29) of the survivors with brain injury were not reported to have adverse long‐term outcome.

## 4 Discussion

### 4.1 Main Findings

This systematic review showed that one in four survivors in MC pregnancies complicated by sFD has brain injury, which was often of antenatal origin in two‐thirds of cases, while postnatal brain injury occurred in the remaining one third. NDI was reported in only one out of 20 long‐term survivors of the overall group. However, among survivors with brain injury, two‐third exhibited NDI, while only one‐third remained unaffected. These rates are lower than previously reported. The latest meta‐analysis of sFD survivors reported brain abnormalities in one in three and NDI in more than one in four survivors. The lower prevalence in our study may be due to several reasons. Firstly, we included more recent literature, allowing for the addition of larger retrospective studies that reduce selection bias seen in smaller case series and small cohort studies [8]. Secondly, literature published before 1999 was not included in this systematic review and meta‐analysis, whereas Mackie et al. predominantly based their analyses (e.g., postnatal brain injury, NDI and NND) on studies published prior to 1999 [8]. Thirdly, the pooled proportions in this review were calculated using strict in‐ and exclusion criteria, thereby excluding certain groups and studies with an a priori increased risk of brain injury or NDI (e.g., cohorts with abnormal scans and case reports ≤ 3 infants). TAPS was excluded because sFD does not lead to brain injury as the minuscule anastomoses preclude acute exsanguination (*of note*: inclusion of TAPS cases would have lowered the pooled proportions even further) [13]. Interestingly, the rate of NND was much lower in our study (6%) compared to previous systematic reviews (28%). Most likely influenced by the exclusion of small case reports with high NND rates and a trend of higher TOP prevalence following sFD in severely affected survivors, as noted in recent studies by Lanna et al. and Klink et al. This trend may be due to advancements in fetal neuroimaging enabling more precise detection of brain injury [3, 4, 30]. Furthermore, improvements in neonatal care, particularly for very/extremely preterm infants, may also have played an important role. We believe that counselling of parents, confronted with sFD, should be done using the most up‐to‐date information to allow them to reach a decision based on more reliable data from the current era.

### 4.2 Strengths and Limitations

This systematic review followed the PRISMA and MOOSE guidelines and was registered with PROSPERO in advance, ensuring transparency in study selection and data extraction [12]. The inclusion of the largest subgroup of MC twin pregnancies, complicated by sFD, provides valuable insights into antenatal and neonatal characteristics, type and prevalence of brain injury, and neurodevelopmental outcomes in this high‐risk population. By including studies published up to 2023, this review integrates the most up‐to‐date evidence and presents current gaps in literature. Despite its strengths, there are several limitations. The heterogeneity was high among included studies, particularly in methodologies, diagnostic criteria for brain injury, availability and quality of neuroimaging and follow‐up duration, complicating comparisons and synthesis of findings, underscoring a high risk for potential selection and publication bias. Another limitation is the inconsistency in imaging methods, with some studies combining pre‐ and/or postnatal imaging, making it challenging to evaluate the timing of brain injury and different types of brain injury occurring at different timepoints. Furthermore, missing data on key variables and the lack of detailed, standardised definitions for specific types of brain injury or NDI may have introduced a bias. Moreover, cases reported before 2010 prior to the formal definition of TAPS may have included undiagnosed cases misclassified under ‘unknown diagnosis’.

### 4.3 Interpretation

An important and novel finding of our study is that survivors with brain injury were born at a lower gestational age at birth, with 62% of them delivered extreme or very preterm. Also, 32% of the brain injury was of postnatal origin. We hypothesize that prematurity could be an important additional risk factor for brain injury and suggest a double‐hit mechanism, that is, the risk of brain injury increases with cumulative exposure to multiple perinatal risk factors [31]. First, acute exsanguination of the surviving co‐twin, potentially causing acute hypoxic–ischemic injury [32, 33, 34]. Followed by a second hit, prematurity‐related brain injury, as most survivors with brain injury are born very/extremely preterm, increasing their susceptibility to additional injury and impaired brain development, and subsequently NDI [35]. A possible third mechanism could be the lack of antenatal corticosteroid and magnesium sulphate administration due to immediate delivery depriving the fetus of neuroprotection [36]. As shown in our study, more than half of surviving twins with brain injury were delivered within a short interval, less than 1 week after sFD, and one‐third even on the same day. Several of these foetuses are thus delivered through emergency caesarean section in an attempt to reduce brain injury, but this could instead increase the risk of (postnatal) brain injury due to (extreme) prematurity and delivery without prior administration of antenatal corticosteroids. Importantly, immediate delivery after sFD is unlikely to reduce the risk of brain injury as the acute event has already occurred [37]. We therefore think that management with immediate delivery after sFD is unwarranted [4, 37, 38, 39, 40]. Importantly, premature delivery by itself can increase the risk of brain injury, NDI, and expectant management may be more beneficial to the infant [41]. Expectant management combined with neuroimaging of the surviving co‐twin (ideally fetal MRI 2–4 weeks after sFD) may be more appropriate [4, 23, 42, 43]. Apparent diffusion coefficient mapping and diffusion‐weighted imaging may help differentiate acute and transient changes for irreversible brain injury. In case neuroimaging rules out brain injury, pregnancy should be continued until term age. If, severe brain injury is detected, TOP or redirection of care at birth could be considered and discussed with parents. However, TOP depends on country‐specific legislation and may not be allowed, particularly when brain injury detected on US or MRI have an uncertain prognosis.

In this systematic review, TTTS and sFGR were the most presumed cause of sFD, however half of the cases remained unexplained [44, 45]. Identifying antenatal risk factors, such as optimal GA at birth, aetiology of sFD, and severity of fetal anaemia following sFD, and the impact of delivery before versus after 32 weeks remains an important area of future research. This highlights the need for larger multicentre studies to refine risk stratification of survivors after sFD [3, 4, 23]. Clinical trials are needed to evaluate the clinical relevance and efficacy of IUT after sFD. Another unresolved question concerns the relationship between the number and diameter of AA and VV anastomoses, and their potential impact on brain injury and NDI [9, 46]. AA and VV anastomoses are low‐resistance bidirectional anastomoses, and larger AA or VV anastomoses may thus allow for larger volumes of exsanguination [3, 47]. To evaluate this hypothesis, future multicentre placental dye injection studies should systematically assess placental vascular architecture in pregnancies complicated by sFD. Furthermore, standardised follow‐up studies should be performed to assess the neurodevelopmental outcome of survivors following sFD, using age‐appropriate cognitive and motor assessments, and thereby enhancing parental counselling and clinical decision‐making.

## 5 Conclusion

This systematic review highlights the significant risk of brain injury and NDI in survivors of MC pregnancies following spontaneous sFD. Our findings suggest a double‐hit injury of survivors with brain injury after sFD, due to the acute exsanguination at the time of sFD, and prematurity‐related brain injury due to the immediate delivery of the survivor after sFD, which may contribute to NDI.

## Author Contributions

M.R. and L.C.M.Z. contributed to conceptualization, methodology, data curation, formal analysis, investigation and drafting of the original manuscript. J.M.M.K., L.S.V., S.J.S. and S.G.G. contributed to methodology, investigation and manuscript review and editing. F.S., E.J.T.(J.)V. and M.C.H. contributed to manuscript review and editing. E.L. contributed to conceptualization, methodology, data curation, formal analysis, investigation, supervision, drafting and review and editing of the manuscript.

## Disclosure

Role of funder/sponsor: The funders had no role in the design and conduct of the study.

## Ethics Statement

The authors have nothing to report.

## Conflicts of Interest

The authors declare no conflicts of interest.

## Supporting information


**Data S1:** bjo70084‐sup‐0001‐DataS1.zip.

## Data Availability

The data that supports the findings of this study is available in the Supporting Information of this article.
