# Supplementary material for: Brain Injury and Neurodevelopmental Outcome in Survivors After Spontaneous Single Fetal Demise in Monochorionic Twins: A Systematic Review and Meta‐Analysis
Source: BJOG. 2025 Nov 12;133(4):570–8. doi: 10.1111/1471-0528.70084 (PMC12884211; doi:10.1111/1471-0528.70084)
Supplement: Supplementary file 1 — Data S1: bjo70084‐sup‐0001‐DataS1.zip. [file BJO-133-570-s001.zip › bjo70084-sup-0001-FigureS1-TableS1-S5-AppendixS1-S2.docx]

**Supplementary Material**

**Figure S1. Funnel plots**
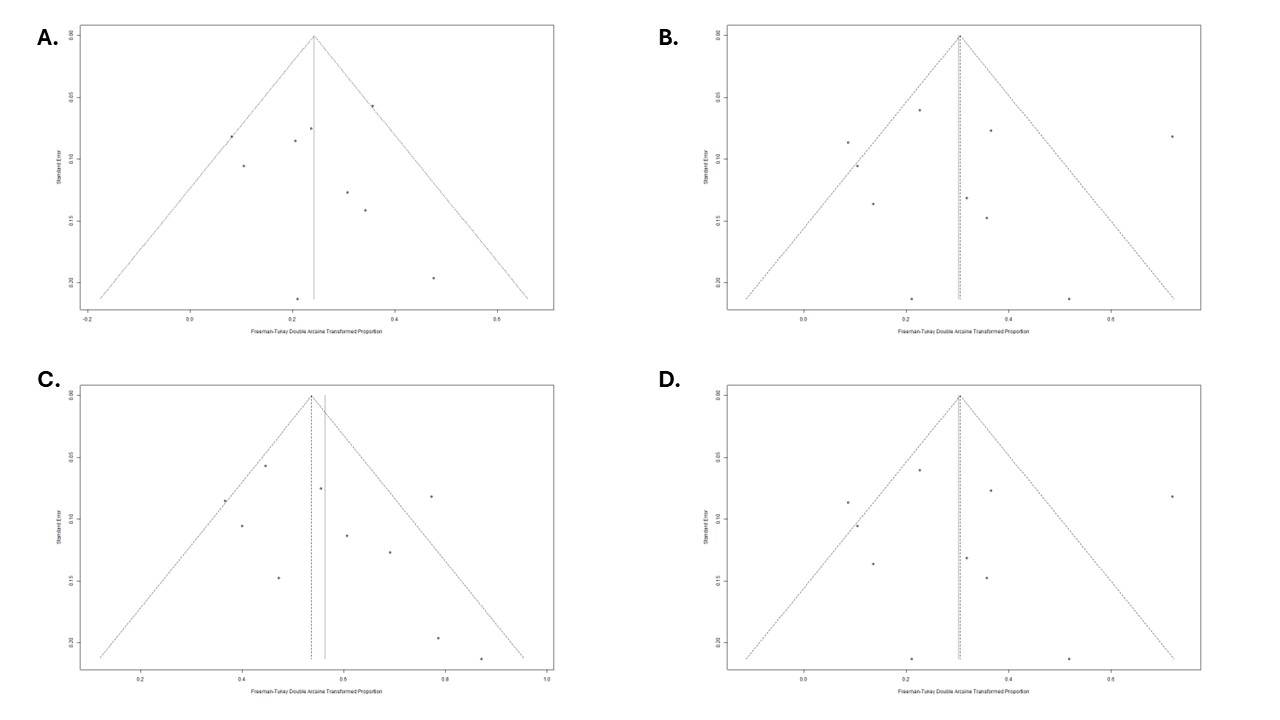


**Table S1. Results Quality Assessment**

| **First author (Year of publication)** | **Selection** | | | | **Comparability** | **Outcome** | | | **Total** |
| --- | --- | --- | --- | --- | --- | --- | --- | --- | --- |
|  | **Representativeness** | **Non-exposed cohort** | **Exposure** | **Outcome of interest** | **Comparability of cohorts** | **Assessment** | **Follow-up** | **Adequacy** |  |
| Duyos et al. (2023) | * | * | * | * | ** | * | * | * | 9/9 |
| Morris et al. (2020) | * | * | * | * | ** | * | * | * | 9/9 |
| Lanna et al. (2020) | * | * | * | * | ** | * | * | * | 9/9 |
| Conte et al. (2018) | * | * | * | - | ** | * | * | * | 8/9 |
| van Klink et al. (2015) | * | * | * | - | ** | * | * | * | 8/9 |
| Jatzko et al. (2015) | - | * | * | * | ** | * | * | - | 7/9 |
| Griffiths et al. (2015) | * | - | * | - | * | * | * | * | 6/9 |
| Tunc et al. (2015) | * | * | * | - | * | * | * | * | 7/9 |
| Hoffmann et al. (2013) | * | * | - | * | * | * | * | * | 7/9 |
| Mahony et al. (2011) | * | * | * | * | * | * | - | * | 7/9 |
| Fichera et al. (2009) | * | * | * | - | ** | * | - | * | 7/9 |
| Jelin et al. (2008) | * | * | * | - | * | * | - | * | 6/9 |
| Bajoria et al. (1999) | * | * | * | - | * | * | - | * | 6/9 |

**Table S2. Baseline characteristics of the included studies**

| First author (year) | Country | Study design | Study period | Number of infants | Population |
| --- | --- | --- | --- | --- | --- |
| Duyos et al. (2023)^23^ | Spain | R | 2012-2020 | 15^a^ | sFD after 14 weeks of GA |
| Morris et al. (2020)^19^ | UK | P | 2016-2017 | 22 | sFD after 14 weeks of GA |
| Lanna et al. (2020)^4^ | Italy | R | 2004-2015 | 77^b^ | sFD after 14 weeks of GA |
| Conte et al. (2018)^22^ | Italy and UK | R | 2002-2015 | 21^c^ | Only survivors with brain injury |
| Klink et al. (2015)^3^ | NL | R | 2002-2013 | 44^d^ | sFD after 12 weeks of GA |
| Griffiths et al. (2015)^24^ | UK | R | 2004-2013 | 34^e^ | sFD after 12 weeks of GA |
| Tunc et al. (2015)^25^ | Turkey | R | 2008-2013 | 13^f^ | sFD after 12 weeks of GA |
| Jatzko et al. (2015)^21^ | Austria | R | 2005-2012 | 5^g^ | sFD after 12 weeks of GA |
| Hoffmann et al. (2013)^20^ | Israel | P | 2007-2010 | 6^h^ | sFD after 12 weeks of GA |
| Mahony et al. (2011)^26^ | Ireland | R | 1997-2006 | 6^i^ | sFD after 24 weeks of GA |
| Fichera et al. (2009)^27^ | Italy | R | 2001-2006 | 12^j^ | sFD after 14 weeks of GA |
| Jelin et al. (2008)^28^ | USA | R | 1997-2007 | 19^k^ | sFD after 12 weeks of GA |
| Bajoria et al. (1999)^29^ | UK | R | 1980-1998 | 37^l^ | sFD after 12 weeks of GA |

**Table S3. Antenatal and neonatal characteristics**

| First author (year) | Ga at demise (weeks) | Ga at birth (weeks) | Aetiology of demise (%) | Top (%) | Nnd (%) | Treated with iuts (%) |
| --- | --- | --- | --- | --- | --- | --- |
| Duyos et al. (2023)^23^ | 22.9 (SD ± 4.1) | 32.6 (SD ± 3.2)   - Moderate preterm 7/13 (54%) - Very preterm 4/13 (31%) - Extreme preterm 1/13 (8%) | 6/15 (40) TTTS,  4/15 (27) sFGR,  2/15 (13) MA,  3/15 (20) Unknown | 1/15 (7) | 1/14 (7) | 4/15 (27) |
| Morris et al. (2020)^19^ | 24.0 (IQR 18.5-29.5) | 35.6 (IQR 33.7-37.5)   - Moderate preterm 16/22 (73%) - Very preterm 4/22 (18%) | NR | 0/22 (0) | 0/22 (0) | NR |
| Lanna et al. (2020)^4^ | 22.0 (range 15.0-36.0) | 36.0 (range 23.0-41.0)   - NR | 30/77 (39) TTTS,  19/77 (25) sFGR,  28/77 (36) Unknown | 9/77 (12) | 3/68 (4) | 2/77 (3) |
| Conte et al. (2018)^22^ | 21.0 (IQR 18.0-24.0) | NR | 6/21 (29) TTTS,  15/21 (71) Unknown | NR | NR | NR |
| Klink et al. (2015)^3^ | 24.0 (IQR 20.0-29.0) | 36.0 (IQR 33.0–38.0)   - NR | 17/44 (39) TTTS,  10/44 (23) sFGR,  5/44 (11) MA,  12/44 (27) Unknown | 2/44 (5) | 5/42 (12) | 6/44 (14) |
| Griffiths et al. (2015)^24^ | 20.9 (IQR 19.0-26.0) | 35.7 (IQR 35.0-38.0)^a^   - Moderate preterm 12/27 (44%) - Very preterm 0/27 (0%) - Extreme preterm 1/27 (4%) | NR | 1/34 (3) | 0/33 (0) | NR |
| Tunc et al. (2015)^25^ | 26.8 (SD ± 5.8) | 34.2 (SD ± 2.5)   - Preterm 6/13 (46%) | 2/13 (15) TTTS,  1/13 (8) sFGR,  10/13 (77) Unknown | NR | 0/13 (0) | NR |
| Jatzko et al. (2015)^21^ | 20.0 (IQR 17.0-23.0) | 38.0 (IQR 30.8-38.5)   - Moderate preterm 1/5 (20%) - Very preterm 1/5 (20%) | NR | 0/5 (0) | 1/5 (20) | NR |
| Hoffmann et al. (2013)^20^ | 29.5 (IQR 26.5-33.0)^b^ | NR   - Preterm 2/4 (50%) | 1/6 (17) Abruptio placentae,  5/6 (83) Unknown | 1/6 (17) | 0/5 (0) | 1/6 (17) |
| Mahony et al. (2011)^26^ | 28.0 (IQR 26.3-32.0) | NR | 4/6 (67) TTTS,  1/6 (17) Abruption placentae,  1/6 (17) Unknown | NR | NR | NR |
| Fichera et al. (2009)^27^ | 24.3 (range 17.0–32.2) | 36.0 (range 28.4–40.2)   - Moderate preterm 6/11 (55%) - Very preterm 2/11 (18%) | 5/12 (42) TTTS^c^,  3/12 (25) sFGR,  4/12 (33) Unknown | 1/12 (8) | 1/11 (9) | 0/12 (0) |
| Jelin et al. (2008)^28^ | 20.0 (IQR 16.5-22.1) | NR | 9/19 (47) TTTS,  4/19 (21) Probable TTTS,  6/19 (32) Unknown | NR | NR | NR |
| Bajoria et al. (1999)^29^ | 28.2 (IQR 26.0-30.1) | 29.0 (IQR 27.4-32.6)   - Moderate preterm 8/37 (22%) - Very preterm 16/37 (43%) - Extreme preterm 10/37 (27%) | 17/37 (46) TTTS,  1/37 (3) sFGR,  19/37 (51) Unknown | 0/37 (0) | 16/37 (43) | 20/37 (54) |
| Overall | 24.0 (IQR 20.5-28.1)* | 35.7 (32.3-37.0)* | TTTS 96/250 (38) sFGR 38/250 (15)  Other 13/250 (5) Unknown 103/250 (41) | 15/252 (6) | 27/250 (11) | 33/191 (17) |

**Table S4. Brain injury and NDI of co-twin survivors after sFD**

| First author (year) | Prevalance of brain injury (%) | Type of ante- or postnatal brain injury (n) | Outcome (n) |
| --- | --- | --- | --- |
| Duyos et al. (2023)^23^ | 6/15^a^ (40) | cPVL (2), encephalomalacia (1), IVH (1), unspecified white matter injury (2) | 2/13 (15%); Normal (2), TOP (1), NND (1), CP (1), global developmental delay (1) |
| Morris et al. (2020)^19^ | 3/22 (14) | Unknown (3) | 2/22 (9%); Normal (1), abnormal neonatal neurological signs (2) |
| Lanna et al. (2020)^4^ | 14/77 (18) | cPVL (4), encephalomalacia (4), cortical malformation (1), infarction (2), volume loss and/or severe ventriculomegaly (2), unspecified white matter injury (1) | 4/65 (6%); Normal (1), TOP (9), severe CP (2), mild neurological morbidity (2) |
| Conte et al. (2018)^22^ | NR | Encephalomalacia (10), unspecified white matter injury (1), unknown (10) | NR |
| Van klink et al. (2015)^3^ | 12/44^b^ (27) | cPVL (2), encephalomalacia (6), IVH (1),  infarction (3) | 5/37 (14%); TOP (2), NND (5), survival (4), CP (2), Delayed motor development (1), tetraplegia (1), severe NDI (1) |
| Griffiths et al. (2015)^24^ | 4/34 (12) | Encephalomalacia (2), unknown (2) | 1/33 (3%); TOP (1), survival (2), Severe NDI (1) |
| Tunc et al. (2015)^25^ | NR | NR | NR |
| Jatzko et al. (2015)^21^ | 3/5 (60) | cPVL (1), encephalomalacia (1), IVH (1) | 1/4 (25%); Normal (1), CP at 5y (1), NND (1) |
| Hoffmann et al. (2013)^20^ | 3/6 (50) | Infarction (2), unknown (1) | 1/5 (20%); Normal (1), TOP (1), motor deficiencies (1) |
| Mahony et al. (2011)^26^ | NR | NR | NR |
| Fichera et al. (2009)^27^ | 2/11 (18) | Infarction (1), unspecified white matter injury (1) | 0/10 (0%); Normal (1), NND (1) |
| Jelin et al. (2008)^28^ | 6/19 (32) | Encephalomalacia (1), cortical malformation (2),  infarction (1), unknown (2) | NR |
| Bajoria et al. (1999)^29^ | 18/37 (49) | cPVL (4), IVH (12), unknown (2) | 2/24 (8%); Normal (3), NND (13), CP and blindness (1), CP (1) |
| Overall | 71/270 (26) | Encephalomalacia (27%, 25/92), IVH (16%, 15/92), cPVL (14%, 13/92), infarctions (10%, 9/92), unspecified WM injury (5%, 5/92), cortical malformations (3%, 3/92), volume loss/severe VM (2%, 2/92) and unknown (19%, 20/92) | NDI: 18/213 (8%) |

**Table S5. Subgroup analysis of survivors after sFD with brain injury**

| Case | Author | GA at Demise (weeks) | GA at Birth (weeks) | Aetiology | Timing of injury | Description of Injury | Neurodevelopmental outcome |
| --- | --- | --- | --- | --- | --- | --- | --- |
| 1 | Duyos et al | 19.0 | NA | sFGR type II | Antenatal | Destructive lesions. Microcephaly | TOP |
| 2 | Duyos et al | 30.0 | 30.2 | sFGR type III | Postnatal | PVL | CP |
| 3 | Duyos et al | 24.6 | 29.0 | sFGR type III | Postnatal | Severe periventricular hyper echogenicity | Normal |
| 4 | Duyos et al | 24.5 | 27.1 | TTTS | Postnatal | PVL | NND |
| 5 | Duyos et al | 19.5 | 33.5 | TTTS | Postnatal | PVL | Global developmental delay |
| 6 | Duyos et al | 24.0 | 28.1 | Cord entanglement MCMA twin | Postnatal | Several periventricular hyper echogenicities | Normal |
| 7 | Morris et al | NR | NR | Spontaneous | NR | CNS abnormality | Abnormal neonatal neurological signs |
| 8 | Morris et al | NR | NR | Spontaneous | NR | CNS abnormality | Abnormal neonatal neurological signs |
| 9 | Morris et al | NR | NR | Spontaneous | NR | CNS abnormality | NR |
| 10 | Lanna et al | 22.3 | 38.0 | TTTS (Donor) | Antenatal | Frontoparietal bilateral PVL | CP and severe neurological morbidity. |
| 11 | Lanna et al | 20.0 | 27.0 | TTTS (Donor) | Antenatal | Irregular lateral ventricles | Mild neurological morbidity |
| 12 | Lanna et al | 21.0 | NA | TTTS (Donor) | Antenatal | Frontal and parietotemporal bilateral ischemia | TOP |
| 13 | Lanna et al | 24.5 | NA | sFGR | Antenatal | Frontal bilateral cortical encephalomalacia | TOP |
| 14 | Lanna et al | 26.0 | NA | sFGR | Antenatal | Parietotemporal bilateral encephalomalacia | TOP |
| 15 | Lanna et al | 22.0 | NA | sFGR | Antenatal | Bilateral PVL | TOP |
| 16 | Lanna et al | 24.0 | NA | Spontaneous | Antenatal | Parietotemporal bilateral ischemia | TOP |
| 17 | Lanna et al | 19.6 | NA | Spontaneous | Antenatal | Parietotemporal bilateral polymicrogyria | TOP |
| 18 | Lanna et al | 15.0 | NA | Spontaneous | Antenatal | bilateral occipital cortical ischemia | TOP |
| 19 | Lanna et al | 18.0 | NA | Spontaneous | Antenatal | Frontoparietal bilateral PVL | TOP |
| 20 | Lanna et al | 17.0 | NA | Spontaneous | Antenatal | bilateral parietooccipital encephalomalacia | TOP |
| 21 | Lanna et al | 20.1 | 25.0 | TTTS (Donor) | Postnatal | IVH grade III | Normal |
| 22 | Lanna et al | 32.0 | 32.0 | sFGR | Postnatal | PVL | Severe neurological morbidity, spastic bilateral CP |
| 23 | Lanna et al | 29.4 | 29.5 | Uncomplicated | Postnatal | PVL | Mild neurological morbidity |
| 24 | Conte et al | 22.0 | NR | TTTS | Antenatal | PVL | NR |
| 25 | Conte et al | 18.0 | NR | Spontaneous | Antenatal | Generalized encephalomalacia | NR |
| 26 | Conte et al | 22.0 | NR | TTTS | Antenatal | Generalized encephalomalacia | NR |
| 27 | Conte et al | 19.0 | NR | Spontaneous | Antenatal | Generalized encephalomalacia | NR |
| 28 | Conte et al | 26.0 | NR | Spontaneous | Antenatal | Generalized encephalomalacia | NR |
| 29 | Conte et al | 17.0 | NR | Spontaneous | Antenatal | Generalized encephalomalacia | NR |
| 30 | Conte et al | 20.0 | NR | Spontaneous | Antenatal | Generalized encephalomalacia | NR |
| 31 | Conte et al | 18.0 | NR | Spontaneous | Antenatal | Posterior encephalomalacia | NR |
| 32 | Conte et al | 17.0 | NR | Spontaneous | Antenatal | Posterior encephalomalacia | NR |
| 33 | Conte et al | 15.0 | NR | Spontaneous | Antenatal | Posterior encephalomalacia | NR |
| 34 | Conte et al | 21.0 | NR | Spontaneous | Antenatal | Posterior encephalomalacia | NR |
| 35 | Conte et al | 28.0 | NR | Spontaneous | Antenatal | bilateral parasagittal or perisylvian lesions | NR |
| 36 | Conte et al | 24.0 | NR | Spontaneous | Antenatal | bilateral parasagittal or perisylvian lesions | NR |
| 37 | Conte et al | 25.0 | NR | TTTS | Antenatal | bilateral parasagittal or perisylvian lesions | NR |
| 38 | Conte et al | 19.0 | NR | Spontaneous | Antenatal | nonhemorrhagic lesions | NR |
| 39 | Conte et al | 17.0 | NR | TTTS | Antenatal | nonhemorrhagic lesions | NR |
| 40 | Conte et al | 19.0 | NR | Spontaneous | Antenatal | nonhemorrhagic lesions | NR |
| 41 | Conte et al | 26.0 | NR | Spontaneous | Antenatal | nonhemorrhagic lesions | NR |
| 42 | Conte et al | 23.0 | NR | TTTS | Antenatal | nonhemorrhagic lesions | NR |
| 43 | Conte et al | 24.0 | NR | Spontaneous | Antenatal | hemorrhagic lesions | NR |
| 44 | Conte et al | 23.0 | NR | TTTS | Antenatal | hemorrhagic lesions | NR |
| 45 | Klink et al | 19.0 | 23.0 | TTTS (Donor) | Antenatal | Right MCA infarction | TOP |
| 46 | Klink et al | 23.0 | 34.0 | TTTS (Donor) | Antenatal | Bilateral MCA infarction | TOP |
| 47 | Klink et al | 28.0 | 37.0 | TTTS (Donor) | Antenatal | Severe cerebral atrophy, diffuse white matter loss, abnormal thalamus and capsula interna | Severe neurodevelopmental delay and CP |
| 48 | Klink et al | 22.0 | 37.0 | TTTS (Donor) | Postnatal | cPVL Grade 3 | Tetraplegia |
| 49 | Klink et al | 26.0 | 27.0 | TTTS (recipient) | Postnatal | Bilateral IVH Grade 3 with PVHI, cPVL Grade 3 | NND |
| 50 | Klink et al | 27.0 | 28.0 | TTTS (recipient) | Postnatal | Unilateral IVH Grade 2, infarction right caudate nucleus | Delayed motor development, tone regulation disorder |
| 51 | Klink et al | 29.0 | 29.0 | TTTS (Donor) | Antenatal | Multicystic encephalopathy | NND |
| 52 | Klink et al | 35.0 | 35.0 | sFGR (larger cotwin) | Postnatal | Diffuse cortical necrosis | Severe NDI |
| 53 | Klink et al | 27.0 | 27.0 | sFGR (larger cotwin) | Postnatal | Bilateral IVH Grade 3 | NND |
| 54 | Klink et al | 31.0 | 31.0 | Spontaneous | Antenatal | Multicystic encephalopathy | Bilateral spastic CP |
| 55 | Klink et al | 36.0 | 36.0 | Spontaneous | Postnatal | Diffuse cortical necrosis, white matter injury and severe basal ganglia/thalamic injury | NND |
| 56 | Klink et al | 36.0 | 36.0 | Spontaneous | Postnatal | Cortical necrosis, white matter injury and severe basal ganglia/thalamic injury | NND |
| 57 | Griffiths et al | 22.0 | 37.0 | Spontaneous | Antenatal | Mild VM | Normal |
| 58 | Griffiths et al | 21.0 | 32.0 | Spontaneous | Antenatal | Mild VM | Normal |
| 59 | Griffiths et al | 17.0 | 38.0 | Spontaneous | Antenatal | VM and microencephaly; Extensive Encephalomalacia, PMG, schizencephaly. | Severe NDI* |
| 60 | Griffiths et al | 20.0 | NA | Spontaneous | Antenatal | VM and microencephaly; extensive encephalomalacia | TOP |
| 61 | Jatzko et al | 20.0 | 29.0 | Spontaneous | Antenatal | IVH grade I | Normal at 1.6y |
| 62 | Jatzko et al | 15.0 | 38.0 | Spontaneous | Antenatal | Cysts lateral to the ganglionic eminence, mild VM/ loss of white matter | CP at 5y |
| 63 | Jatzko et al | 25.0 | 32.6 | Spontaneous | Antenatal | IVH III, cerebral edema, laminar necrosis, extensive parenchymal defects | NND 2 days after birth |
| 64 | Hoffmann et al | 36.0 | NA | Spontaneous | Antenatal | Brain edema; recent posterior infarct | TOP |
| 65 | Hoffmann et al | 32.0 | 36.0 | Spontaneous | Antenatal | Contralateral previous infarct | Motor deficiencies 1 year |
| 66 | Hoffmann et al | 25.0 | 37.0 | Spontaneous | Antenatal | Hydrops | Normal |
| 67 | Fichera et al | 28.4 | 28.4 | Spontaneous | Antenatal | Severe IVH, bilateral ventriculomegaly, periventricular hemorrhage | NND 3 days after birth |
| 68 | Fichera et al | 31.4 | 31.5 | Spontaneous | Postnatal | Mild VM and periventricular hyperechogenicities | Normal at 12 months |
| 69 | Jelin et al | 15.5 | NR | TTTS | Antenatal | Unilateral infarct with developing PMG | NR |
| 70 | Jelin et al | 17.5 | NR | TTTS | Antenatal | Focal injury in left parietal lobe with dilation of adjacent ventricle | NR |
| 71 | Jelin et al | 20.0 | NR | Spontaneous | Antenatal | Bilateral mild VM; delayed sulcation | NR |
| 72 | Jelin et al | 21.0 | NR | TTTS | Antenatal | Sylvian fissures slightly shallow for age | NR |
| 73 | Jelin et al | 21.6 | NR | TTTS | Antenatal | Extensive encephalomalacia | NR |
| 74 | Jelin et al | 20.0 | NR | TTTS | Antenatal | Bilateral choroid plexus and germinal matrix hemorrhage with VM; posterior fossa hemorrhage subarachnoid hemorrhage near the cerebellum. | NR |
| 75 | Bajora et al | 27.1 | 27.1 | Spontaneous | NR | IVH | NND |
| 76 | Bajora et al | 28.1 | 28.4 | Spontaneous | NR | IVH | NND |
| 77 | Bajora et al | 27.0 | 27.0 | Spontaneous | NR | IVH | NND |
| 78 | Bajora et al | 28.3 | 28.3 | Spontaneous | NR | cyst | NND |
| 79 | Bajora et al | 30.1 | 30.1 | Spontaneous | NR | IVH, PVL | CP and blindness |
| 80 | Bajora et al | 28.2 | 35.0 | Spontaneous | NR | IVH | CP |
| 81 | Bajora et al | 27.1 | 27.3 | Spontaneous | NR | IVH, PVL | NND |
| 82 | Bajora et al | 28.0 | 28.0 | Spontaneous | NR | IVH | NND |
| 83 | Bajora et al | 28.4 | 30.2 | sFGR | NR | IVH, PVL | NND |
| 84 | Bajora et al | 31.0 | 32.0 | Spontaneous | NR | IVH | Normal |
| 85 | Bajora et al | 28.3 | 29.0 | Spontaneous | NR | IVH | Normal |
| 86 | Bajora et al | 28.0 | 28.0 | sFGR | NR | IVH. Cyst | NND |
| 87 | Bajora et al | 25.0 | 25.0 | sFGR | NR | IVH | NND |
| 88 | Bajora et al | 25.0 | 25.1 | sFGR | NR | IVH, PVL, cyst | NND |
| 89 | Bajora et al | 26.0 | 26.0 | sFGR | NR | IVH | NND |
| 90 | Bajora et al | 24.6 | 25.1 | sFGR | NR | IVH, cyst | NND |
| 91 | Bajora et al | 29.0 | 29.0 | sFGR | NR | IVH | NND |
| 92 | Bajora et al | 28.5 | 34.2 | Spontaneous | NR | Porencephalic cyst | Normal |

**Appendix S1. Search strategy**

The search strategy will include the search terms:

***Pubmed***

Combination of #1 AND #2 NOT #4

(“monochorionic*”[tiab] OR “mono-chorion*”[tiab] OR “MC Pregnan*”[tiab] OR “MC Twin*”[tiab] OR “MCDA”[tiab] OR “MCMA”[tiab] OR “Identical Twin*”[tiab] OR “single placenta”[tiab] OR “shared placenta”[tiab] OR “common placenta”[tiab]) AND (“fetal demise”[tiab] OR “foetal demise”[tiab] OR “Fetal Death”[Mesh] OR “Fetal Death*”[tiab] OR “foetal death*”[tiab] OR “Stillbirth*”[tiab] OR “Stillbirth”[Mesh] OR “Stillborn”[tiab] OR “Intrauterine death*”[tiab] OR “Perinatal death*”[tiab] OR “Perinatal Loss”[tiab] OR “IUFD”[tiab] OR “IUFDs”[tiab] OR ((“Mortality”[Mesh] OR “Mortality”[tiab] OR “Death”[Mesh] OR “Death”[tiab] OR “Demise”[tiab] OR “Loss”[tiab]) AND (“Fetus”[tiab] OR “Foetal”[tiab] OR “fetal*”[tiab] OR “prenatal”[tiab] OR “Antenatal”[tiab]))) NOT (“case report*”[pt] OR “case report*”[ti] OR “Editorial”[pt] OR “Letter”[pt] OR “Editorial”[ti] OR “Letter”[ti] OR “review”[pt])

Results: 711

***Web of science***

(((TI=(("monochorionic" OR "mono-chorion" OR "mono-chorionic" OR "MC Pregnancy" OR "MC Pregnancies" OR "MC Twin" OR "MC Twins" OR "MCDA" OR "MCMA" OR "Identical Twin" OR "Identical Twins" OR "single placenta" OR "shared placenta" OR "common placenta") )) AND TS=(("fetal demise" OR "foetal demise" OR "Fetal Death" OR "Fetal Deaths" OR "foetal death" OR "foetal deaths" OR "Stillbirth" OR "Stillborn" OR "Intrauterine death*" OR "Perinatal death*" OR "Perinatal Loss" OR "IUFD" OR "iucds" OR ((Mortalities OR Mortality OR Death OR Demise OR Loss) AND (Fetus OR Foetal OR fetal* OR prenatal OR Antenatal))) )) NOT SO=(("case report" OR "case reports" OR "Editorial" OR "Letter" OR "review"))) NOT TI=(("case report" OR "case reports" OR "Editorial" OR "Letter") )

Results: 429

***Embase***

("monochorionic*".ti. OR "mono-chorion*".ti. OR "MC Pregnan*".ti. OR "MC Twin*".ti. OR "MCDA".ti. OR "MCMA".ti. OR "Identical Twin*".ti. OR "single placenta".ti. OR "shared placenta".ti. OR "common placenta".ti.) **AND** ("fetal demise".ti,ab. OR "foetal demise".ti,ab. OR "Fetus Death"/ OR "Fetal Death*".ti,ab. OR "foetal death*".ti,ab. OR "Stillbirth*".ti,ab. OR exp "Stillbirth"/ OR "Stillborn".ti,ab. OR "Intrauterine death*".ti,ab. OR "Perinatal death*".ti,ab. OR "Perinatal Loss".ti,ab. OR "IUFD".ti,ab. OR "IUFDs".ti,ab. OR ((exp "Mortality"/ OR "Mortality".ti,ab. OR exp "Death"/ OR "Death".ti,ab. OR "Demise".ti,ab. OR "Loss".ti,ab.) AND ("Fetus".ti,ab. OR "Foetal".ti,ab. OR "fetal*".ti,ab. OR "prenatal".ti,ab. OR "Antenatal".ti,ab.)))

Results: 1877

***Scopus***

TITLE-ABS ("monochorionic*" OR "mono-chorion*" OR "MC Pregnan*" OR "MC Twin*" OR "MCDA" OR "MCMA" OR "Identical Twin*" OR "single placenta" OR "shared placenta" OR "common placenta") AND TITLE-ABS ("fetal demise" OR "foetal demise" OR "Fetal Death" OR "foetal death" OR "Stillbirth*" OR "Stillborn" OR "Intrauterine death*" OR "Perinatal death*" OR "Perinatal Loss" OR "IUFD" OR "IUFDs" OR ("Mortality" OR "Death" OR "Demise" OR "Loss") AND ("Fetus" OR "Foetal" OR "fetal*" OR "prenatal" OR "Antenatal"))

Results: 1232

**Appendix S2**

Quality assessment of non-randomized studies included in this systematic review

**Newcastle-Ottawa Scale**Selection
1) Representativeness of the exposed cohort
a) Truly representative (one star)
b) Somewhat representative (one star)
c) Selected group
d) No description of the derivation of the cohort

2) Selection of the non-exposed cohort
a) Drawn from the same community as the exposed cohort (one star)
b) Drawn from a different source
c) No description of the derivation of the non-exposed cohort

3) Ascertainment of exposure
a) Secure record (e.g., surgical record) (one star)
b) Structured interview (one star)
c) Written self-report
d) No description
e) Other

4) Demonstration that outcome of interest was not present at start of study
a) Yes (one star)
b) No

Comparability (tick one or both boxes, as appropriate)
1) Comparability of cohorts on the basis of the design or analysis controlled for confounders
a) The study controls for age, sex, and marital status (one star)
b) Study controls for other factors (list) ___________ (one star)
c) Cohorts are not comparable on the basis of the design or analysis controlled for confounders

Outcome
1) Assessment of outcome
a) Independent blind assessment (one star)
b) Record linkage (one star)
c) Self report
d) No description
e) Other

2) Was follow-up long enough for outcomes to occur
a) Yes (one star)
b) No
Indicate the median duration of follow-up and a brief rationale for the assessment above:____________________

3) Adequacy of follow-up of cohorts
a) Complete follow up- all subject accounted for (one star)
b) Subjects lost to follow up unlikely to introduce bias- number lost less than or equal to 20% or description of those lost suggested no different from those followed. (one star)
c) Follow up rate less than 80% and no description of those lost
d) No statement

Thresholds for converting the Newcastle-Ottawa scales to AHRQ standards (good, fair, and poor):

Good quality: 3 or 4 stars in selection domain AND 1 or 2 stars in comparability domain AND 2 or 3 stars in outcome/exposure domain

Fair quality: 2 stars in selection domain AND 1 or 2 stars in comparability domain AND 2 or 3 stars in outcome/exposure domain

Poor quality: 0 or 1 star in selection domain OR 0 stars in comparability domain OR 0 or 1 stars in outcome/exposure domain
