# Supplementary material for: Brain Injury and Neurodevelopmental Outcome in Survivors After Spontaneous Single Fetal Demise in Monochorionic Twins: A Systematic Review and Meta‐Analysis
Source: BJOG. 2025 Nov 12;133(4):570–8. doi: 10.1111/1471-0528.70084 (PMC12884211; doi:10.1111/1471-0528.70084)
Supplement: Supplementary file 1 — Data S1: bjo70084‐sup‐0001‐DataS1.zip. [file BJO-133-570-s001.zip › Table Legends.docx]

**Table Legends**

**Table S1 Results Quality Assessment**Results of the quality assessment of the studies using the Newcastle-Ottawa scale. Abbreviations: G, good quality, F, fair Quality, P, poor quality, ⋆, one star.

**Table S2. Baseline characteristics of the included studies**Abbreviations: DC, dichorionic; dFD, double fetal demise; FLS, fetal laser surgery; GA, gestational age; MC, monochorionic; NL, Netherlands; P, prospective cohort study; R, retrospective cohort study; sFD, single fetal demise; TRAP, twin reversed arterial perfusion; UK, United Kingdom; USA, United States of America. ^a^ One case with a malformation was excluded. ^b^ One case with dFD was excluded. ^c^ Twenty-one cases of selective feticide, FLS, first-trimester demise, and multi-order pregnancies were excluded. ^d^ One multi-order pregnancy, two DC pregnancies, and two cases with congenital anomalies were excluded. ^e^ Four cases with unknown time of sFD, one case with dFD, and two cases with first-trimester demise were excluded. ^f^ Six cases with first-trimester demise were included. ^g^ Six cases with sFD after FLS were excluded. ^h^ Twenty-eight cases with FLS or selective feticide were excluded. ^i^ Five cases of dFD were excluded. ^j^ One case with TTTS, in which FLS was performed, was included and one case with dFD was excluded. ^k^ Two cases involving TRAP were excluded. ^l^ Thirteen cases of dFD were excluded.

**Table S3. Antenatal and neonatal characteristics**Abbreviations: FLS, fetal laser surgery; GA, gestational age; IQR, interquartile range; IUT, intrauterine transfusion; MA, monoamniotic twin; NND, neonatal death; NR, not reported; SD, standard deviation; sFD, single fetal demise; sFGR, selective fetal growth restriction; TOP, termination of pregnancy; TTTS, twin-to-twin transfusion syndrome. ^a^ Seven cases with one TOP and six cases in which it was not reported were excluded. ^b^ In two cases the GA at demise was unknown. ^c^ One TTTS case treated with FLS was included. *Summary of medians and IQRs, excluding studies with SD or range.

**Table S4. Brain injury and NDI of co-twin survivors after sFD**

Abbreviations: CP, cerebral palsy; cPVL, cystic periventricular leukomalacia; FLS, fetal laser surgery; IVH, intraventricular haemorrhage; NDI, neurodevelopmental impairment; NND, neonatal death; NR, not reported; TOP, termination of pregnancy; UCO, umbilical cord occlusion; VM, ventriculomegaly, WM, white matter. ^a^ Seven cases of sFD after FLS or UCO were excluded. ^b^ One multi-order pregnancy was excluded.

**Table S5. Subgroup analysis of survivors after sFD with brain injury**Overview of all cases of survivors after sFD in MC pregnancies, including GA at demise and birth, neurodevelopmental outcome, description of injury and aetiology. Abbreviations: NR, not reported, NND, neonatal death, GA, gestational age, sFGR, selective fetal growth restriction, TTTS, twin-to-twin transfusion syndrome, MCMA, monochorionic monoamniotic, PVL, periventricular leukomalacia, IVH, intraventricular haemorrhage, VM, ventriculomegaly, PMG, polymicrogyria, CP, cerebral palsy, cPVL, cystic periventricular leukomalacia, TOP, termination of pregnancy. *Given the severity of this case with encephalomalacia, polymicrogyria, and microcephaly, we classified this as severe NDI, as no NDI would be inappropriate.
